# Protocol for *in vivo* lineage tracing of regeneration-associated macrophages from injured skeletal muscle of adult mice

**DOI:** 10.1016/j.xpro.2025.103844

**Published:** 2025-05-24

**Authors:** Neuza S. Sousa, Pedro Sousa-Victor, Joana Neves

**Affiliations:** 1 GIMM- Gulbenkian Institute for Molecular Medicine, 1649-035 Lisbon, Portugal; 2 Faculdade de Medicina, Universidade de Lisboa, 1649-028 Lisbon, Portugal

**Keywords:** Cell Biology, Cell isolation, Flow Cytometry, Immunology, Model Organisms

## Abstract

Macrophages undergo phenotypic transitions that are essential for successful skeletal muscle (SkM) regeneration. Here, we present a protocol for *in vivo* lineage tracing of regeneration-associated macrophages, combining genetic labeling with transplantation of fluorescence-activated cell sorting (FACS)-isolated cells. Macrophages isolated from congenic CD45.1^+^ donor mice are transplanted into pre-injured SkMs of CD45.2^+^ mice and phenotyped by flow cytometry at designated time points of the regenerative process. We describe steps for muscle injury, SkM tissue processing, macrophage isolation, transplantation, and flow cytometry phenotyping.

For complete details on the use and execution of this protocol, please refer to Sousa et al.^1^

## Highlights

• Injury and conditioning of skeletal muscle of donor and recipient mice for transplant
• Instructions for isolating regeneration-associated macrophages by FACS
• Steps for transplanting individual macrophage subpopulations into recipient muscles
• Procedures for phenotyping transplanted macrophages in recipient muscles

## Abstract (editor highlights)

Publisher’s note: Undertaking any experimental protocol requires adherence to local institutional guidelines for laboratory safety and ethics.

## Graphical abstract

**Figure:**
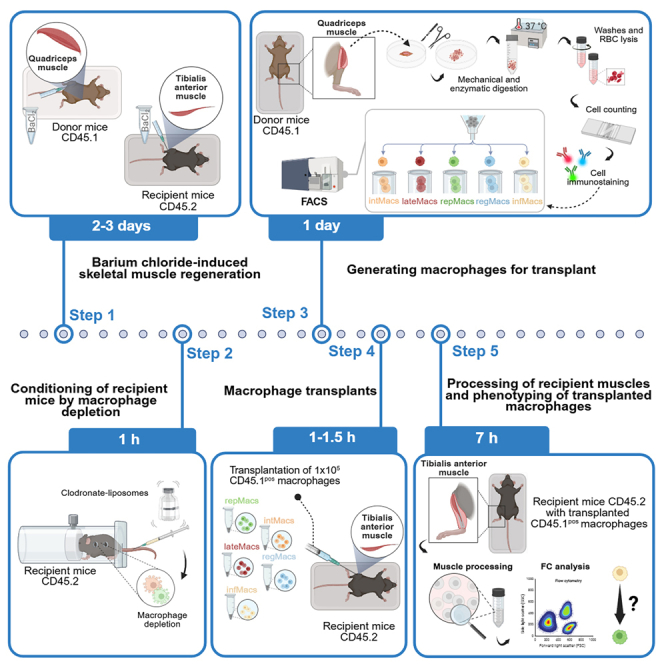

## Before you begin

This protocol can be used to assess the differentiation trajectories of macrophages during the process of skeletal muscle (SkM) regeneration. Regeneration-associated macrophages derive from infiltrating monocytes that differentiate within the muscle into macrophages, and undergo a stereotyped process of phenotypic transitions whereby Ly6C^High^ populations give rise to heterogeneous Ly6C^Low^ states.^1^^,^^2^^,^^3^ Perturbations to the process of macrophage phenotypic transition have been implicated in regenerative failure.^3^^,^^4^^,^^5^ We have recently characterized the heterogeneity of the macrophage compartment throughout the regenerative process using single-cell RNA sequencing, identifying different macrophage populations that participate in the regenerative process and are differentially affected by aging.^1^ During SkM regeneration, macrophages transition through distinct functional states. In the early phase, infiltrating monocyte-derived macrophages (infMacs) and regeneration-associated regulatory macrophages (regMacs) are the predominant macrophage states. infMacs and regMacs express high levels of *Ly6c2*, but while infMacs exhibit classical inflammatory and tissue-infiltrating gene signatures, regMacs also express genes linked to immune suppression and chemoattraction. Both infMacs and regMacs can be identified by flow cytometry as Ly6C^High^MHCII^neg^, and distinguished based on the expression of CD109, whereby regMacs are CD109^pos^ and infMacs are CD109^neg^. In the later phases of SkM regeneration, *Ly6c2*-low macrophages become predominant. Repair-associated macrophages (repMacs) peak at 3 dpi and are characterized by genes involved in growth factor signaling, lipid metabolism, and lysosomal activity. Late regeneration-associated macrophages (lateMacs), accumulate at later stages and show a transcriptional signature enriched for antigen presentation and interferon response genes. Both repMacs and lateMacs can be identified by flow cytometry as Ly6C^low^, and distinguished based on the expression of CD109 and MHC II, whereby repMacs are MHCII^neg^CD109^pos^ and lateMacs are Ly6C^low^MHCII^pos^CD109^neg^. A transition state of intermediate macrophages (intMacs) express intermediate levels of Ly6c2, co-express markers of both repMacs and lateMacs, and can be identified by flow cytometry as Ly6C^high^MHCII^pos^CD109^pos+neg^ and Ly6C^low^MHCII^pos^CD109^pos^ (See ref.^1^ for more details).

We have applied this protocol to trace the differentiation trajectories of regeneration-associated macrophages in healthy young mice.^1^ This protocol can also be used to investigate defects in phenotypic transition in conditions of SkM regenerative failure, such as aging and myopathies, to study the contribution of specific factors to the process of phenotypic transition, and adapted to study other macrophage subpopulations identified in the SkM.^6^^,^^7^

Our macrophage lineage tracing assay is based on the transplantation of regeneration-associated macrophage populations, isolated by fluorescence-activated cell sorting (FACS) from injured SkM of CD45.1^+^ donor mice, into pre-injured SkM of recipient CD45.2^+^ wild-type (WT) recipient mice (C57BL/6). Recipient mice undergo chemical macrophage ablation one day prior to transplantation. Adoptive transfers are done between time-matched injured animals. A characterization of the transplanted CD45.1^pos^ population in the recipient SkM (16 h after transplant) allows the identification of the macrophage subpopulations arising from the original transplanted subpopulation.

### Institutional permissions

All mice used in this work were housed at the Direção Geral da Alimentação e Veterinaria (DGAV) accredited rodent facility of GIMM – Gulbenkian Institute for Molecular Medicine. All mouse experiments reported in this study complied with relevant institutional and national animal welfare laws, guidelines and policies. The experimental protocol was reviewed and approved by Orgão de Bem Estar e Ética Animal do Institutode Medicina Molecular ( iMM-ORBEA) and licensed by Direcção Geral de Alimentação e Veterinária (DGAV, project license number 022860∖2020).

All mouse experiments must be conducted with the approval of the animal care committee at your research institution.

### Preparation of 1.2% barium chloride solution

**Timing: 20 min**

1. Prepare barium chloride (BaCl_2_) solution.
  a. Dissolve BaCl_2_ in sterile saline solution (0.9% NaCl) to make a 1.2% BaCl_2_ working solution.
  b. Filter BaCl_2_ working solution through a 0.22 μm filter and store at −20°C in 600 μL aliquots (40 μL, or 50 μL, will be injected per tibialis anterior (TA) or quadriceps (QC) muscle, respectively).

**CRITICAL:** It is important to maintain sterile conditions throughout this process.

### Preparation of buffers and media

**Timing: 10–20 min**

2. Prepare Wash medium by supplementing DMEM medium with 1% Penicillin/Streptomycin (Pen/Strep).
3. Prepare Complete medium.
  a. Add 10% fetal bovine serum (FBS) and 1% Pen/Strep into DMEM medium.

***Optional:*** Filter the complete medium through a Stericup vacuum filtration system (pore size 0.22 μm).

4. Prepare FACS buffer by adding 5% Horse serum (HS) to Dulbecco’s phosphate-buffered saline (DPBS).

***Optional:*** In the preparation of FACS buffer, 5% HS can be substituted with 5% FBS.

## Key resources table

**Table:** 

REAGENT or RESOURCE	SOURCE	IDENTIFIER
**Antibodies**		

APC anti-mouse CD209a (DC-SIGN; clone MMD3) (1:20)	BioLegend	Cat#833005; RRID: AB_2927969
APC anti-mouse NK-1.1 (clone PK136) (1:25)	BioLegend	Cat#108710; RRID: AB_313396
APC anti-mouse CD170 (Siglec-F; clone S17007L) (1:80)	BioLegend	Cat#155508; RRID: AB_2750236
APC-eFluor 780 anti-mouse CD11b (clone M1/70) (1:200)	Invitrogen (eBioscience)	Cat#47-0112-82; RRID: AB_1603193
Pacific Blue anti-mouse I-A/I-E (clone M5/114.15.2) (1:200)	BioLegend	Cat#107620; RRID: AB_493527
Brilliant Violet 711 anti-mouse Ly-6C (clone HK1.4) (1:40)	BioLegend	Cat#128037; RRID: AB_2562630
FITC anti-mouse Ly-6G (clone 1A8) (1:400)	BioLegend	Cat#127606; RRID: AB_1236488
PE anti-human/mouse CD109 (clone # 496920) (1:10)	Bio-Techne/R&D Systems	Cat#FAB4385P; RRID: AB_10643402
PE-Cyanine5 anti-mouse CD45.1 (clone A20) (1:500)	Invitrogen (eBioscience)	Cat#15-0453-82; RRID: AB_468759

**Chemicals, peptides, and recombinant proteins**		

Barium chloride	Sigma-Aldrich	Cat#202738-5G
Dulbecco’s modified Eagle’s medium (DMEM)	Corning	Cat#10-013-CMR
Penicillin and streptomycin	Thermo Fisher Scientific (Gibco)	Cat#15140122
Collagenase B	Roche	Cat#11088815001
Calcium chloride (CaCl_2_) solution 2.5 M	Jena Bioscience	Cat#BU-103
RBC lysis buffer (10X)	Santa Cruz Biotechnology	Cat#sc-296258
UltraPure DNase/RNase-free distilled water	Thermo Fisher Scientific (Invitrogen)	Cat# 10977035
Fetal bovine serum (FBS)	Sigma	Cat#F9665
Horse serum (HS)	Sigma	Cat#H1138
Clodronate liposomes (5 mg mL^−1^)	LIPOSOMA	SKU #CP-010-010
Isoflurane ISOVET 250 mL	B. Braun	Cat#469860
Sterile saline solution (NaCl 0.9%) 5 mL	B. Braun	Cat#3653800
Dulbecco’s phosphate-buffered saline (DPBS)	Thermo Fisher Scientific (Gibco)	Cat#14190144
IC fixation buffer	Thermo Fisher Scientific (Invitrogen)	Cat#2865823
Trypan blue, 0.4%	Thermo Fisher Scientific (Gibco)	Cat#15250061

**Experimental models: Organisms/strains**		

Mouse: C57BL/6 (males and females)	Charles River	Strain #027; RRID:IMSR_CRL:027
Mouse: CD45.1^+^/Ly5.1 (males and females)	Charles River	Strain #002014; RRID: IMSR_JAX:002014

**Software and algorithms**		

FlowJo v.10.10	BD Biosciences	RRID:SCR_008520
GraphPad Prism 9	GraphPad Software	RRID:SCR_002798

**Other**		

70 μm cell strainers	Corning	Cat#431751
Centrifuge for 50 mL tubes	Calservice Heratec,S.L.	Cat#GZ-1248R
Rotor of centrifuge for 50 mL tubes	Calservice Heratec,S.L.	Cat#GRS-G-r250-4
Centrifuge for 1.5 mL tubes	Calservice Heratec, S.L.	Cat#GZ-1730R
Aspirator with trap flask	Fisher Scientific, Lda	Cat#15241166
VWR LA Classic, analytical balance	VWR Collection	Cat#611-3343
Hemocytometer	VWR	Cat#630-2183
Standard insulin syringe with needle Sol-M 1 mL 1/2 in 29G	Sol-Millennium Medical	Cat# #1612912B
Safety tuberculin syringe with needle Sol-Care 1 mL 1/2 in 27G retractable safety needle regular wall	Sol-Millennium Medical	Cat# #100019IM
Polypropylene conical tube, 15 mL	VWR	Cat#CORN430790
Polypropylene conical tube, 50 mL	VWR	Cat#CORN430828
5 mL round bottom polystyrene tubes, with cell strainer snap cap	Falcon	Cat#352235
1.5 mL Eppendorf tubes	VWR	Cat# BITXMTL-0150-BC
Petri dishes, 90 mm	Normax	Cat#1.6502055
Memmert basic series water bath	Fisher Scientific	Cat# 10573693
Stericup quick release-GP sterile vacuum bottle top filtration systems	MilliporeSigma	Cat#S2GPU05RE
Guarded scalpel, individually wrapped	VWR	Cat#SWAN6607
Curved fine scissors	Fine Science Tools	Cat#91461-11
Spring scissors	Fine Science Tools	Cat#91500-09
Dumont #5 forceps	Fine Science Tools	Cat#11252-30
BD FACSAria III cell sorter	BD Biosciences	N/A
BD FACSAria Fusion cell sorter	BD Biosciences	N/A
BD LSRFortessa X-20 cell analyzer	BD Biosciences	N/A
SomnoFlo low-flow electronic vaporizer	Kent Scientific Corporation	Cat#*SF-01*
Sliding top chamber for small animals	Kent Scientific Corporation	Cat#SOMNO-0530SM
SurgiSuite multi-function surgical platform	Kent Scientific Corporation	Cat#SURGI-M
Aesculap Isis rodent shaver	AgnTho’s AB	Cat# GT421

## Materials and equipment

**Table:** Wash medium

Reagent	Volume (mL)	Final concentration
DMEM medium	495 mL	–
Penicillin-Streptomycin (100X)	5 mL	1%
**Total**	**500 mL**	–

***Note:*** Store at 4°C for a maximum of 1 month.

**Table:** Complete medium

Reagent	Volume (mL)	Final concentration
DMEM medium	445 mL	–
Penicillin-Streptomycin (100X)	5 mL	1%
Fetal bovine serum	50 mL	10%
**Total**	**500 mL**	–

***Note:*** Store at 4°C for a maximum of 1 month.

**Table:** FACS buffer

Reagent	Volume (mL)	Final concentration
Horse serum (HS)	1 mL	5%
DPBS	19 mL	–
**Total**	**20 mL**	–

***Note:*** Store at 4°C for a maximum of 1 week.

**Table:** Muscle digestion media (volume for one sample)

Reagent	Amount	Final concentration
Wash medium	5 mL	–
Collagenase B (0.24U/mg)	10 mg	0.2%
CaCl_2_ 2.5 M	1 μL	0.5 mM
Total	**5 mL**	–

***Note:*** Prepare just before using. Warm the wash medium at 37°C before dissolving the Collagenase B. Prepare this solution for all samples together by multiplying the amount of each component by the number of samples. Keep the prepared solution at 23°C–26°C until use and make sure you do not have precipitation of collagenase B.

**CRITICAL:** Collagenase B powder is harmful if inhaled.

**Table:** Red blood cell lysis buffer (volume for one sample)

Reagent	Volume (mL)	Final concentration
UltraPure DNase/RNase-Free Distilled Water	4.5 mL	–
RBC lysis buffer (10 X)	500 μL	1 X
**Total**	**5 mL**	–

***Note:*** Prepare fresh before using.

**Table:** Fixation buffer

Reagent	Volume (mL)	Final concentration
FACS Buffer	100 μL	–
Intracellular (IC) Fixation Buffer	100 μL	1:2
**Total**	**200 μL**	–

***Note:*** Prepare fresh before using.

**CRITICAL:** Intracellular (IC) Fixation Buffer contains Paraformaldehyde, which may cause allergic skin reactions.

## Step-by-step method details

This section lists the major steps to perform *in vivo* lineage tracing of macrophages during SkM regeneration.

### Barium**-**chloride-induced skeletal muscle regeneration

**Timing: 2–3 days (depends on the collection time of the injured muscle)**

To induce muscle regeneration, we applied a chemical injury by intramuscular injection of barium chloride (BaCl_2_). BaCl_2_ administration as a method of muscle injury induces myonecrosis in muscle tissue by eliciting prolonged depolarization of myofibers.^8^

For this protocol, we performed muscle injury of quadriceps (QC, donor mice) and tibialis anterior (TA, recipient mice) muscles. Both donor and recipient mice muscles are injured on the same day to match the timing of their time-point of the regenerative process. The larger size of the QC muscle maximizes the number of isolated macrophages for transplant, while using a smaller muscle (TA) as recipient minimizes the diffusion, allowing for an optimized activity and recovery of the transplanted cells, minimizing the number of mice used.

***Note:*** For an estimate of the number of mice required for your experiment, based on the number of macrophages of each subtype isolated from one QC at 2- and 3-days post injury (dpi) refer to Table 1. Consider that each recipient mouse receives 1 × 10^5^ macrophages. When necessary (e.g. the first time performing the experiment or after equipment maintenance), an additional injured mouse, preferably a CD45.1^+^ mouse, should be used to generate the necessary single-color controls for setting up the cell sorter or flow cytometer.

**Figure 1:**
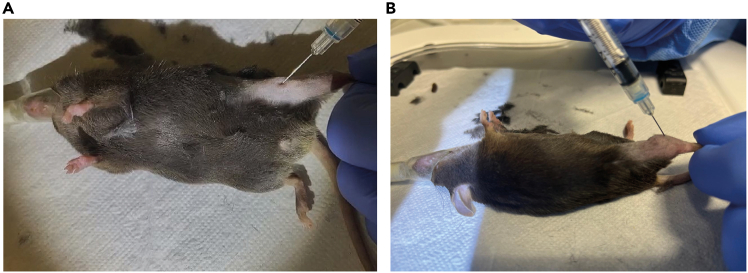
Mouse intramuscular injections Mouse intramuscular injection on quadriceps (A) or tibialis anterior (B) muscle
    ii. For the TA injury, insert the needle perpendicular to the TA belly and then reduce the angle to 45º relative to the fibula for the tip of the needle to be in the mid belly of the TA and slowly inject 40 μL of 1.2% BaCl_2_ (See Figure 1B).
      **CRITICAL:** Be careful when inserting the needle to ensure it does not go beyond the muscle. If the technique is performed properly, no bleeding should occur. If bleeding is observed after the injection it may indicate a rupture of a blood vessel and the animal should not be included in the experiment.
  e. Slowly remove the needle from the muscle and dispose in a sharps bin.
    ***Note:*** Removing the needle too quickly can lead to leakage.
  f. Return the mouse to its cage. Keep it under observation to confirm it has fully recovered from anesthesia before leaving it unattended.
    ***Note:*** It is difficult to assess the efficiency of muscle injury before dissection. Nevertheless, one way to confirm the effective delivery of BaCl_2_ solution into the muscle is by observing an expansion of the tissue during the injection and a slight transparency in the muscle region when exposed to direct light.
  g. Allow muscle regeneration to proceed to 2 or 3 days before performing the macrophage transplants.

1. Muscle injury by intramuscular injection of 1.2% BaCl_2_ of donor and recipient mice.
  a. Prior to injection, take out aliquots of BaCl_2_ working solution to equilibrate to 23°C–26°C (∼20 min).
    ***Note:*** Aliquots of BaCl_2_ working solution are stored at −20°C.
  b. Anesthetize each mouse by using an appropriate induction chamber with an SomnoFlo Low-Flow Electronic Vaporizer of isoflurane.
    i. Place the mouse inside the chamber and close the lid.
    ii. Adjust isoflurane vaporizer setting to gas flow of 500 mL/min and 5% of isoflurane.
      ***Note:*** Adjust the flow rate depending on the induction chamber size you are using.
    iii. Wait until the mouse becomes deeply unconscious and then remove the mouse from the chamber.
      ***Note:*** Complete anesthesia can be confirmed by loss of pedal reflex.
      **CRITICAL:** Do not overexpose the mouse to the isoflurane, as excessive exposure can lead to death.
    iv. After purging the chamber (gas flow 1000 mL/min for 10s), take the mouse and place it on a heating pad, maintaining anesthesia by using gas flow of 50 mL/min with 2% of isoflurane provided via a mask.
      ***Note:*** To prevent hypothermia, place the mouse on a heating pad during the process.
  c. Shave the hair of the hindlimb where the TA or QC muscle will be injected to expose the skin over the specific muscle.
  d. Using a 1 mL syringe with a 27-gauge needle, inject 1.2% BaCl_2_ into the QC muscle (50 μL) or TA (40 μL) of the mice.
    i. For QC injury, insert the needle parallel to the femur into the QC mid belly and slowly inject 50 μL of 1.2% BaCl_2_ per QC (See Figure 1A).

**Table 1:** Expected numbers of individual macrophage populations (infMacs, regMacs, intMacs, repMacs or lateMacs) and sorting for one injured quadriceps from a 2- to 3-month old female mouse, at 2 and 3 dpi

Macrophage subpopulation	2 days post injury	Sorting time	3 days post injury	Sorting time
infMacs	15 × 10^4^–23 × 10^4^ cells	2 h–2.5 h	15 × 10^4^–25 × 10^4^ cells	1.5 h–2 h
regMacs	1 × 10^5^–17 × 10^4^ cells		16 × 10^4^–18 × 10^4^ cells	
intMacs	5 × 10^4^–1 × 10^5^ cells		15 × 10^4^–2 × 10^5^ cells	
repMacs	9 × 10^4^–16 × 10^4^ cells		35 × 10^4^–55 × 10^4^ cells	
lateMacs	2 × 10^4^–3 × 10^4^ cells		5 × 10^4^–8 × 10^4^ cells	

The number of cells can vary depending on the age and sex of the mice, and level of muscle injury. This table gives an estimate of what to expect, allowing better planning of the experiments. Sorting time is influenced by the presence of debris in our sample.

### Conditioning of recipient mice by macrophage depletion

**Timing: ∼1 h (depends on the number of mice to be injected)**

To prepare the injured muscle for transplantation, macrophages must be depleted a day prior to prevent overpopulation following transplantation. This is achieved by administering clodronate-liposomes intravenously. We delivered the liposomes via tail vein injection.

***Note:*** Liposome solution could also be injected intravenously from the retro-orbital sinus.

2. Intravenous injection of clodronate-liposomes.
  a. Prepare clodronate-liposomes for injection.
    i. Prior to injection, take out clodronate-liposomes to equilibrate to 23°C–26°C (∼30 min).
      ***Note:*** Clodronate liposomes are stored at 4°C.
    ii. Immediately before injection, invert the liposome tube several times (8–10 times) until the liquid becomes homogenized.
    iii. Use a 1 mL insulin syringe with a 29-gauge needle to take the appropriate dose of clodronate-liposomes from the vial.
      **CRITICAL:** Make sure to remove any air bubbles before injecting the mouse. Injecting air into the vein can lead to death.
  b. Prepare mice for tail vein injection.
    i. Weigh the mice. Based on the weight, the volume of clodronate-liposomes to be injected into each mouse is calculated at a dose of 100 μL per 10 g of body weight.
    ii. Position the mouse cages at least 15 cm from a heat lamp, for 10–15 min, to enable tail vein dilation.
      **CRITICAL:** Prevent mice from becoming overheated.
    iii. Place the mouse inside a restrainer to facilitate a lateral tail vein injection in conscious mice.
    iv. Position the mouse (inside the restrainer) on its right or left side.
  c. Inject clodronate liposomes through the tail vein.
    i. Hold the tail with your non-dominant hand and stretch it into a straight line.
      ***Note:*** The tail vein should be visible as a purple, straight line facing up.
    ii. Insert needle tip into the tail vein at a 10º angle. The needle should slide very easily once you are inside the vein.
    iii. Slowly inject the volume of clodronate-liposomes into the lateral tail vein.
      **CRITICAL:** The injection must feel smooth, and a short whitening of the tail vein must be observed. If resistance is felt while injecting and if a whitening of the area around the injection site can be observed, intravenous injection was not successful.
    iv. After injection, wait 30 s with the needle still inside the vein.
    v. Once the injection is completed, place the mouse back into its cage.

### Generating macrophages for transplant

**Timing: ∼1 day**

Injured QC muscles from donor mice are dissected, and a mechanical and enzymatic digestion of the muscle is performed to obtain single-cell suspensions. A flow cytometry panel designed to identify regeneration-associated macrophage subpopulations (see Sousa et al., 2024)^1^ is used to isolate each population by FACS.

**Figure 2:**
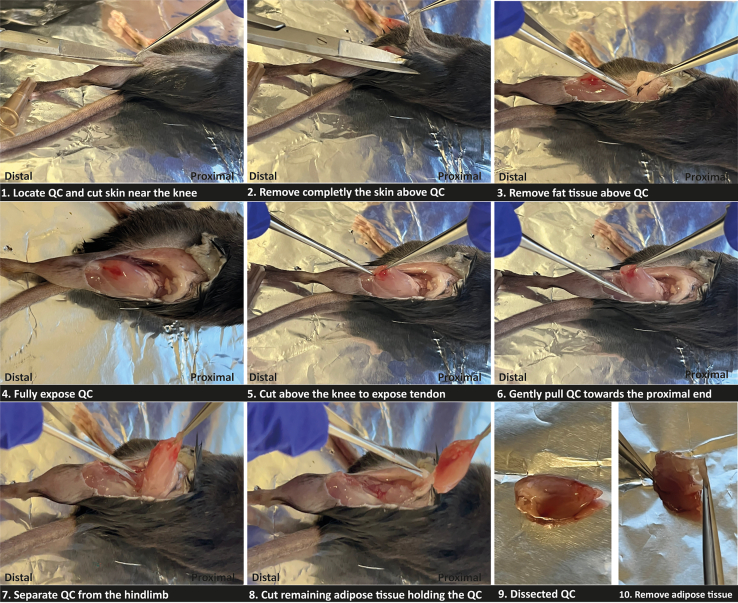
Step-by-step procedure for quadriceps muscle dissection from mice
6. Mechanically process the dissected muscle.
  a. Place the muscle in a petri dish containing 100 μL of complete medium.
    i. Mince the muscle into small pieces, using curved surgical scissors.
    ii. Further mince the muscle with a scalpel.
  b. Collect the minced muscle, using a 10 mL pipette filled with 5 mL of cold complete medium, to a 50 mL tube.
    i. Wash the petri dish of remaining minced muscle with 5 mL of complete medium and use a razor blade to help.

***Note:*** This step can be repeated one more time. The maximum volume in the 50 mL collection tube is 15 mL.

***Note:*** Repeat steps 4–6 for each muscle. It is recommended to disinfect the dissection area between mice with 70% ethanol to avoid cross-contamination of samples. Use different petri dishes and 50 ml tubes to collect each sample.

3. Prepare materials for muscle dissection and processing.
  a. Before starting muscle dissection, prepare all necessary media and materials.
    i. Prepare muscle digestion media containing 0.2% Collagenase B and keep it at 23°C–26°C until step 7c.
    ii. Pre-cool the centrifuges for 50 mL and 1.5 mL tubes and pre-cool all buffers.
    iii. Keep wash medium and complete medium on ice during all processing.
      ***Note:*** Samples should be kept always on ice.
  b. Prepare a de-contaminated dissection area on a benchtop by cleaning it with 70% ethanol.
  c. Prepare and label a tube of 50 mL to collect each muscle. Keep the tubes on ice.
4. Euthanize the mouse according to your institution’s approved protocol.
5. Dissect the injured QC muscles (See Figure 2).
  a. After the mouse is sacrificed, immediately lay it on a dissection board on its back.
    i. Position the hindlimb in order to obtain a frontal view of the QC muscle.
    ii. Stabilize the hands and feet of the mouse with needles.
  b. Spray the hindlimb of the mouse with 70% EtOH.
  c. Carefully pinch the outer skin of the hindlimb and gently cut and remove the skin and fascia covering the QC to reveal the underlying muscle, using fine-tip forceps and micro surgical scissors.
  d. Use the forceps to pick the QC and spring scissors to detach the muscle above the knee.
    i. Carefully cut the muscle along the femur towards its proximal end until the muscle detaches.
    ii. Make a cut at the proximal end of the muscle to fully detach it from the bone.
    iii. Use the forceps to remove any remaining non-muscle tissue.
  e. Place the muscle in a small weighing boat.
    ***Note:*** When processing muscles from more than five mice, it is advisable to have multiple people collaborating, as managing this large number of samples can be time-consuming.
  f. Rapidly, weigh the dissected muscle and register the value.
    ***Note:*** Weighing the dissected muscle is necessary to normalize the total number of cells obtained from the processed muscle, allowing the assessment of effective injury.

**CRITICAL:** Keep the samples on ice while other samples are prepared, ensuring that all samples are digested simultaneously.

7. Digest injured QC muscles.
  a. Centrifuge samples at 400 × g for 5 min at 4°C.
  b. Carefully remove supernatant (floating fat is normally observed and needs to be removed) using a 10 mL pipette.
    **CRITICAL:** Be careful to avoid aspirating the muscle pellet, as it is typically somewhat loose.
  c. Resuspend the muscle pellet with muscle digestion media. Incubate the samples at 37°C for 1 h, using a water bath.
    **CRITICAL:** Shake vigorously every 10 min.
    ***Note:*** At the end of the incubation period, verify the persistence of visible pieces of muscle. If that occurs, the incubation can be extended for 20 min (maximum).
8. Stop the digestion reaction and wash the digested muscle.
  a. Decant the digested muscle through a 70 μm cell strainer placed in a new 50 mL tube.
    i. Use 10 mL of complete medium to wash the walls of the digestion tube and the cell strainer.
      ***Note:*** Washing the digested muscle with ice cold complete medium stops the digestion. A second filtration through a 70 μm cell strainer to a clean 50 mL tube can be performed if needed.
  b. Centrifuge samples at 670 × g for 10 min at 4°C.
  c. Remove the supernatant using an aspirator.
  d. Resuspend the pellet in 5 mL of complete medium.
    i. First resuspend the pellet in 1 mL of complete medium by pipetting up-and down.
    ii. Lastly, add the remaining 4 mL of complete medium.
      ***Note:*** Step necessary to remove traces of collagenase B.
  e. Centrifuge samples at 670 × g for 10 min at 4°C.
  f. Remove the supernatant using an aspirator.
    ***Note:*** Use the aspirator to clean the 50 mL tube walls of liquid droplets.
9. Perform red blood lysis.
  a. Resuspend (pipet up and down) each pellet in 500 μL of 1x RBC lysis buffer.
  b. Incubate on ice for 10 min.
    ***Note:*** Protect from light.
    **CRITICAL:** Do not incubate for more than 10 min. Excessive incubation time can lead to an increase in cell death.
  c. Add 9 mL of wash medium to stop red blood lysis.
  d. Centrifuge samples at 670 × g for 10 min at 4°C.
  e. Discard the supernatant by decantation and use the aspirator to clean the walls of liquid droplets.
    ***Note:*** Pellet should appear white, without blood cells present. RBC lysis can be repeated until a clear white pellet is visible.
10. Resuspend the pellet in 1 mL of complete medium and count the cells on a hemocytometer.

***Note:*** For an injured QC muscle, 3–7 × 10^4^ cells/mg should be expected at 2 dpi and 7–12 × 10^4^ cells/mg at 3 dpi (See troubleshooting-problem 1).

***Optional:*** Verify cell viability by performing trypan blue staining during cell counting.

11. Stain cells for sorting.
  a. Resuspend experimental muscle samples in the master mix of fluorescence-conjugated antibodies.
    i. Prepare the master mix of fluorescence-conjugated antibodies in FACS buffer and keep it on ice and protected from light until use.
      **CRITICAL:** Prepare the master mix for all samples, considering 100 μL per 1 × 10^6^ cells. Add the fluorescence-conjugated antibodies according to the designated antibody dilutions presented in Table 2.
      ***Note:*** We use antibody staining to positively select CD45.1^pos^ and CD11b^pos^ cells and negatively exclude dendritic cells CD209^pos^, eosinophils (SiglecF^pos^), Natural killer cells (NK1.1^pos^) and neutrophils (Ly6G^pos^), as dendritic cells can express MHCII and eosinophils, natural killer cells and neutrophils can express Ly6C contaminating the macrophages populations. Then, we use cell surface markers, Ly6C, CD109 and MHCII, previously shown to distinguish different regeneration-associated macrophage subpopulations (regMacs, infMacs, intMacs, repMacs, lateMacs).^1^

**Table 2:** Antibody panel for FACS

Fluorophore	Antibody target	Final dilution
APC	CD209a (DC-SIGN)	1:20
APC	NK1.1	1:25
APC	CD170 (SiglecF)	1:80
APC-eFluor 780	CD11b	1:200
Pacific Blue	IA-IE (MHCII)	1:200
Brilliant Violet 711	Ly6C	1:40
FITC	Ly6G	1:400
PE	CD109	1:10
PE-Cyanine5	CD45.1	1:500
    ii. Transfer the cell suspension into a 1.5 mL tube and centrifuge at 500 × g for 5 min at 4°C.
    iii. Aspirate the supernatant and resuspend the pellet in the master mix at a density of 1 × 10^6^ cells/100 μL.
  b. Prepare extra cells for single-color controls and fluorescence-minus-one (FMO) controls to perform settings on the cell sorter.
    ***Note:*** This step only needs to be performed on the first cell sorting session, or if otherwise instructed by the cell sorter operator due to instrument maintenance interventions.
    i. Transfer 5 × 10^5^ cells from a processed injured muscle to 1.5 mL tubes.
    ii. Perform the stain of each control by resuspending the cells in 50 μL of FACS buffer with the respective antibodies (dilutions presented in Table 2).
      ***Note:*** Single-colors controls are prepared for each antibody used individually. FMO controls are prepared by adding all but one antibody, for each color used.
  c. Incubate cells (experimental samples for FACS and controls) on ice for 30 min, protected from light.
    ***Note:*** Before incubation with the master mix, Fc blocking should be used to block nonspecific binding of the Fc receptors.
  d. Centrifuge the cells at 500 × g for 5 min at 4°C.
  e. Aspirate the supernatant and resuspend the pellet in FACS buffer.
    i. Resuspend the samples for FACS at a density of approximately 15 × 10^6^ cells/mL.
    ii. Resuspend single-colors and FMO controls in 200 μL of FACS buffer.
12. Prepare samples, controls and collection tubes before sorting.
  a. Prepare 1.5 mL tubes with 500 μL of complete medium for collection of each macrophage subpopulation from each cell sample and leave them on ice.
  b. Immediately before starting the sorting, filter the cells (experimental samples for FACS and controls) through the cell strainer cap of the FACS tube to avoid clumps.

***Note:*** Keep the cells on ice and protected from light.

**Figure 3:**
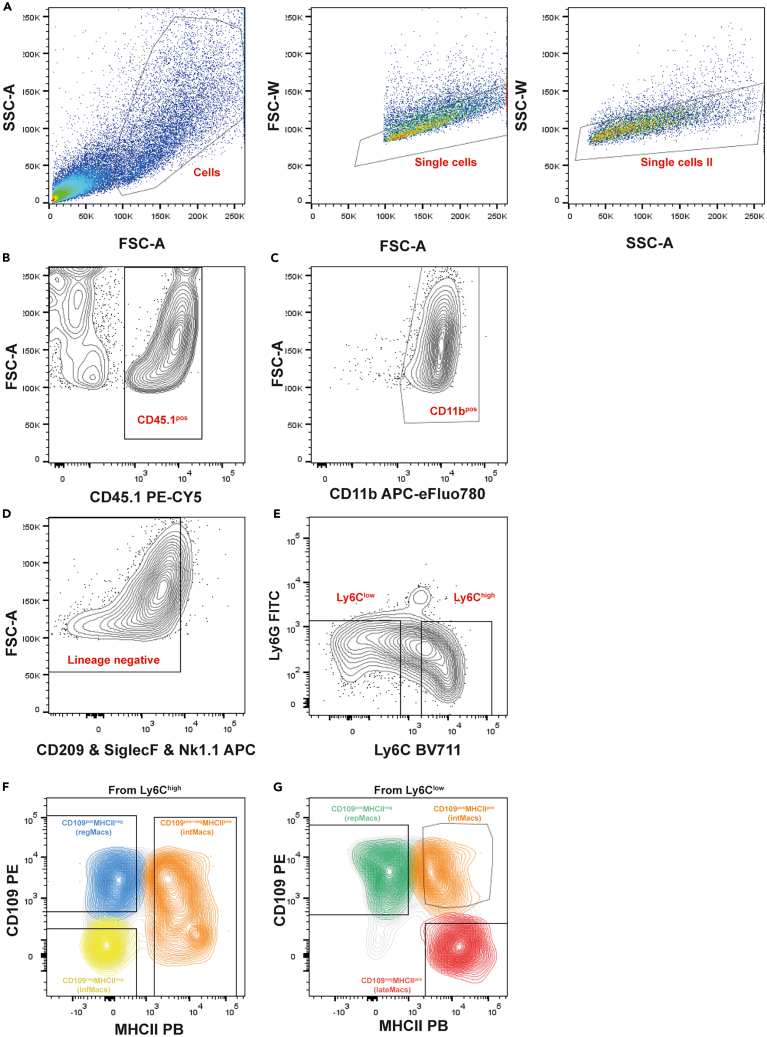
Representative gating strategy for sorting of regeneration-associated macrophage subpopulations (regMACS, infMacs, intMacs, repMacs, lateMacs) This representative sample was obtained from muscles at 2 dpi. (A) Debris removal (SSC-Area vs. FSC-Area) and gating of single cells (FSC-Width vs. FSC-Area-Single cells; and SSC-Width vs SSC-Area-Single cells II). (B) Gating of CD45.1^pos^ cells. (C) Gating of CD11b^pos^ cells. (D) Exclusion of dendritic cells CD209^pos^, Eosinophils, NK1.1 cells by negative selection markers. (E) Exclusion of Ly6G^pos^ cells (neutrophils) by negative selection followed by Ly6C^high^ and Ly6C^low^ gating for the two main macrophage populations. (F and G) Identification of macrophage subpopulations within the Ly6C^high^ and Ly6C^low^ gates, based on the expression of CD109 and MHCII. The identified populations include infMacs (Ly6C^high^MHCII^neg^CD109^neg^), regMacs (Ly6C^high^MHCII^neg^CD109^pos^), repMacs (Ly6C^low^MHCII^neg^CD109^pos^), intMacs (Ly6C^high^MHCII^pos^CD109^pos+neg^ and Ly6C^low^MHCII^pos^CD109^pos^), and lateMacs (Ly6C^low^MHCII^pos^CD109^neg^).

13. FACS of regeneration-associated macrophage subpopulations from single-cells of injured muscle (See troubleshooting-problems 2, 3, and 4).
  ***Note:*** This procedure is typically carried out by a core facility or by personnel with prior expertise. The general gating strategy used is detailed in Figure 3.
  a. Define the settings on the cell sorter.
    i. Use the Forward and Side scatter (FSC-Area vs SSC-Area) to exclude debris and exclude the doublets (FSC-Width vs FSC-Area and SSC-Width vs SSC-Area).
    ii. Run your unstained control to set the background voltage for each channel.
    iii. Run each single-color control sample and set voltage for positive cells and record events.
    iv. Perform automatic compensation matrix calculations for all channels.
    v. Record events for a small amount of sample so that the appropriate gating strategy can be established.
      ***Note:*** At this point, manual compensation may be necessary to make adjustments.
    vi. Run FMO samples to assist with drawing negative gates for APC (CD209, SiglecF, NK.1.1) and Ly6G, and for gating the populations CD45.1^pos^, CD11b^pos^, Ly6C^high^ and Ly6C^low^, CD109^pos^ and CD109^neg^ and MHCII^pos^ and MHCII^neg^. See representative gating strategy in Figure 3.
  b. Sort each population into a 1.5 mL tube, previously prepared with 500 μL of complete medium and keep on ice (maximum 42 × 10^4^ cells per 1.5 mL tube, considering the nozzle and volume in the collecting tubes being used).
    i. Run the sorting until you obtain the desired numbers of each macrophage population.
      ***Note:*** In this protocol, cell sorting was performed using the BD FACSAria III and FACSAria Fusion cell sorter. The following sorting parameters were applied: an 85 μm nozzle, 4-way purity precision, a sheath fluid pressure of 45 PSI, and a threshold rate of 2000 to 5000 events/s. In Table 1, we provide the estimated number for each macrophage population (infMacs, regMacs, intMacs, repMacs or lateMacs) that can be isolated from one donor mouse and the associated sorting time.
      **CRITICAL:** 1.5 mL collecting tubes must be placed in a cooled (4°C) collection tube holder during sorting to maintain the cell viability. During the sorting of other samples, maintain the sorted samples on ice.
  c. Check the purity of the sorted cells by running a small amount of the sorted sample and recording events (ideally >95% purity).
    ***Note:*** The purity of some macrophage subpopulations may be suboptimal due to the initiation of the phenotypic transition during the time of sorting (∼90% purity).
  d. Once all samples are sorted, proceed with the preparation of cells for transplantation.
    ***Note:*** At this stage, the sorted cells can be used for alternative downstream applications, such as *ex vivo* assays or qPCR analysis.

### Macrophage transplants

**Timing: ∼1–1.5 h (approximately 5 min to transplant cells into a recipient mouse; total time depends on the number of recipient animals to be transplanted)**

Individual macrophage populations (infMacs, regMacs, intMacs, repMacs or lateMacs), isolated by FACS from 2 or 3 dpi QC muscles of CD45.1^+^ donor mice, are injected into the TA muscle of a CD45.2^+^ recipient mouse, previously injured at a matching time point, and depleted of macrophages.

14. Prepare the sorted cells for transplant.
  ***Note:*** These steps should be performed inside the laminar flow cabinet, to maximize sterility. Maintain the sorted cells on ice or 4°C during all steps, to preserve cell viability.
  a. Centrifuge the sorted cells at 500 × g for 5 min at 4°C.
  b. Carefully aspirate the supernatant without disturbing the cell pellet.
  c. Pool each sorted population of cells from all donor animals by collecting the pellets in 200 μL of saline.
  d. Centrifuge the pooled cells at 500 × g for 5 min at 4°C.
  e. Based on the number of each sorted population, resuspend the cells using sterile saline solution, to a concentration of 3 × 10^3^ cells/μL.
    ***Note:*** The resuspension volume should be determined based on the total number of cells after pooling samples (e.g., for a FACS count of 12 × 10^4^ cells, resuspend the pooled cell pellet in 40 μL of sterile saline).
  f. Prepare individual aliquots for transplant in 1.5 mL tubes containing 40 μL of cell suspension (12 × 10^4^ cells).
    ***Note:*** The goal is to inject 1 × 10^5^ cells/TA muscle, however we prepare samples with an excess of cells to account for cell loss during injection.
  g. Store the cell suspension on ice until transplantation.
15. Intramuscular injection of individual macrophage populations into the TA muscle.
  a. Proceed as described in step 1b.
  b. If necessary, shave again the hair of the hindlimb where the TA will be injected, to expose the skin over the muscle.
  c. Immediately before transplanting the cells, carefully resuspend the cells by mixing them with a pipette.
  d. Carefully take 40 μL of the resuspended cells with a 1 mL insulin syringe with a 29-gauge needle.
    **CRITICAL:** Avoid air bubbles.
  e. For the intramuscular injection in the pre-injured TA, insert the needle perpendicular to the TA belly and then reduce the angle to 45º relative to the fibula for the tip of the needle to be in the mid belly of the TA and slowly inject 40 μL of cell suspension.
    **CRITICAL:** Be careful when inserting the needle to ensure it does not go beyond the muscle.
  f. Slowly remove the needle from the TA and dispose in a sharps bin.
    ***Note:*** Removing the needle too quickly can lead to leakage of volume from the TA.
  g. Return the mouse to its cage and observe the mouse to confirm it has fully recovered from anesthesia before leaving it unattended.
16. Mice are left in the cage for a designated time. In our experience, 16 h are sufficient to observe the phenotypic transition of the transplanted macrophages.

### Processing of recipient muscles

**Timing: ∼6 h**

At the designated time after transplantation, the recipient TA muscle from WT CD45.2^+^ mice is collected and processed for the characterization of the transplanted CD45.1 population by flow cytometry.

**Figure 4:**
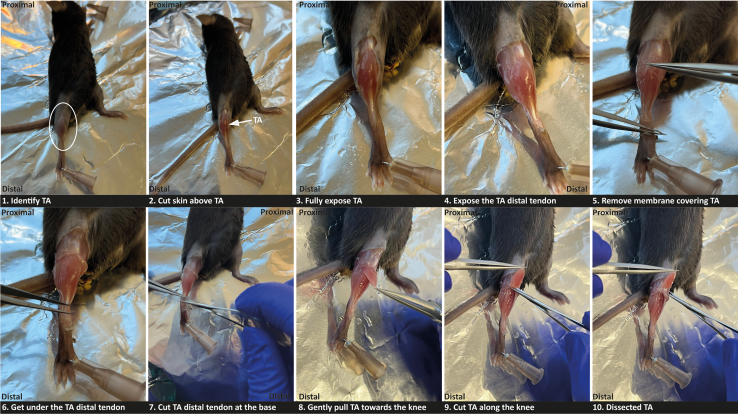
Step-by-step procedure for tibialis anterior (TA) muscle dissection from mice
19. Mechanically process the dissected muscle
  a. Perform step 6a.
  b. Collect the minced muscle, using a 10 mL pipette filled with 3 mL of cold complete medium, to a 50 mL tube.
    i. Wash the petri dish of remaining minced muscle with 2 mL of complete medium and use a razor blade to help.

***Note:*** This step can be repeated one more time. The maximum volume in the 50 mL collection tube is 10 mL.

***Note:*** Repeat steps 17–19 for each muscle. It is recommended to disinfect the dissection area between mice with 70% ethanol to avoid cross-contamination of samples. Use different petri dishes and 50 ml tubes to collect each sample.

17. Perform steps 3 and 4.
18. Dissect recipient TA muscles (See Figure 4).
  a. After the mouse is sacrificed, immediately lay it on the dissection board on its back.
    i. Position the hindlimb in order to obtain a frontal view of the TA muscle.
    ii. Stabilize the hands and feet of the mouse with needles.
  b. Spray the hindlimb of the mouse with 70% EtOH.
  c. Carefully pinch the outer skin of the hindlimb and make an incision to remove the skin and fascia covering the TA muscle, exposing the underlying tissue.
    ***Note:*** Use fine-tip forceps and microsurgical scissors for precise dissection.
  d. Using the forceps, gently grasp the epimysium covering the TA muscle and carefully strip it away along the fibula.
  e. Secure the distal tendon of the TA muscle with forceps and excise it using spring scissors.
  f. While holding the tendon with forceps, gently lift the TA muscle toward the knee to facilitate its detachment.
  g. Cut the proximal tendon as close to the knee as possible to fully isolate the muscle.
  h. Perform steps 5e-f.

**CRITICAL:** Keep the samples on ice while preparing other samples, ensuring they are digested simultaneously.

20. Perform step 7.
21. Stop the digestion reaction and wash the digested muscle.
  a. Decant the digested muscle through a 70 μm cell strainer placed in a new 50 mL tube.
    i. Use 5 mL of complete medium to wash the walls of the digestion tube and the cell strainer.
      ***Note:*** Washing the digested muscle with ice cold complete medium stops the digestion. A second filtration through a 70 μm cell strainer to a clean 50 mL tube can be performed if necessary.
  b. Perform steps 8b-c.
  c. Resuspend the pellet in 2.5 mL of complete medium as in step 8d.
  d. Perform steps 8e-f.
    ***Note:*** Use the aspirator to clean the 50 mL tube walls of liquid droplets.
22. Perform steps 9 and 10.

***Note:*** For recipient muscles, 35 × 10^3^–7 × 10^4^ cells/mg should be expected at 3 dpi (injected with macrophages at 2 dpi) and 4 × 10^4^–75 × 10^3^ total cells/mg at 4 dpi (injected with macrophages at 3 dpi) (See troubleshooting-problem 1).

23. Stain cells from experimental samples, single-color and FMO controls for flow cytometry analysis following steps 11a–d.

***Note:*** For the flow cytometry analysis, use the whole sample of muscle from the recipient mouse, as the percentage of CD45.1^pos^ cells transplanted is minimal compared to the CD45.2^pos^ cells in the host muscle.

24. Fix the stained sample and prepare for flow cytometry analysis.
  a. Aspirate the supernatant and fix the cells by resuspending them in 200 μL of fixative solution. Incubate for 30 min at 23°C–26°C, or 16 h at 4°C.
  b. Wash cells by adding 100 μL of FACS buffer.
  c. Centrifuge cells at 500 × g for 5 min at 4°C.
  d. Resuspend the cells in 300 μL of FACS buffer and store at 4°C protected from light until ready to run on the flow cytometer.
    ***Note:*** Fixed cells should be stable for up to 1 week.
  e. Immediately before analyzing the samples on the flow cytometer, filter the cells through the cell strainer cap of the FACS tube to avoid clumps.

### Phenotyping of transplanted macrophages

**Timing: ∼1 h (depending on the number of samples to be analyzed)**

This section outlines the characterization of the CD45.1^pos^ transplanted macrophages in the recipient muscle. The following steps detail the protocol for identifying the subpopulations derived from the originally transplanted cells within the recipient SkMs, after a designated time post-transplantation.

**Figure 5:**
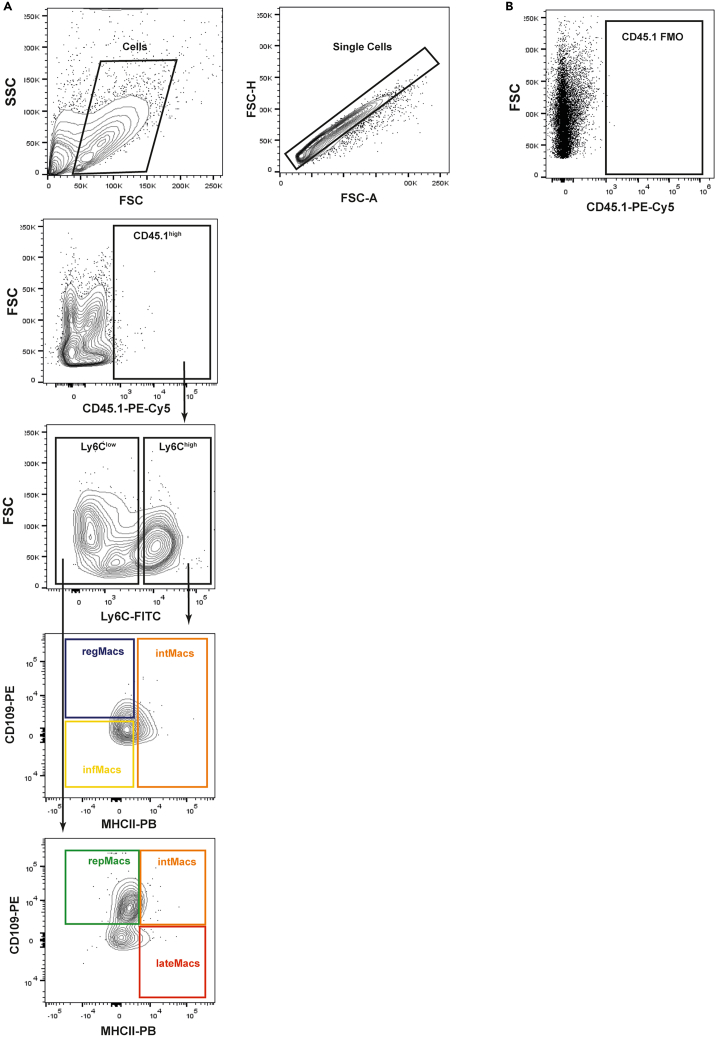
Gating strategy for flow cytometry analysis using FlowJo software Representative plots show the recommended gating strategy to identify the regeneration-associated macrophage subpopulations (regMacs, infMacs, intMacs, repMacs and lateMacs) derived from transplanted CD45.1^pos^ macrophages. (A) Gating strategy for the FC analysis of macrophage subpopulations during SkM regeneration, gated on CD45.1^pos^CD11b^pos^SiglecF/CD209/NK1.1/Ly6G^low^cells: infMacs (Ly6C^high^MHCII^neg^CD109^neg^), regMacs (Ly6C^high^MHCII^neg^CD109^pos^), repMacs (Ly6C^low^MHCII^neg^CD109^pos^), intMacs (Ly6C^high^MHCII^pos^CD109^pos+neg^ and Ly6C^low^MHCII^pos^CD109^pos^), and lateMacs (Ly6C^low^MHCII^pos^CD109^neg^). (B) FMO density plot used to define the CD45.1^pos^ population in the FC panel.
  b. Run the samples on the flow cytometer and record events.

25. Using a flow cytometer, record the CD45.1^pos^ transplanted population of macrophages (See troubleshooting-problems 4 and 5).
  ***Note:*** In this protocol, conventional flow cytometry was performed using the BD LSRFortessa X-20 Cell Analyzer.
  a. Define the settings on the flow cytometer following step 13a.
    ***Note:*** The gating strategy and representative data are shown below (Figure 5). For more information about FMOs used to isolate the different macrophage subpopulations see Sousa et al.^1^

## Expected outcomes

The outcome of this protocol is a quantification of the lineage transition events associated with each macrophage subpopulation during SkM regeneration. Following flow cytometry analysis, the number of CD45.1^pos^ cells expected to be recovered for analysis is in a range of 1 × 10^3^–5 × 10^3^ cells. The breakdown of the CD45.1^pos^ population reveals how many of the transplanted macrophages have retained their original identity or transitioned into a different state. In a young healthy animal, analyzed at 2 dpi, it is expected that: (i) more than 50% of infMacs transition into other states, including regMacs (10%), repMacs (29%), intMacs (22%) and late Macs (2%); (ii) intMacs transition into Ly6C^Low^ states only (repMacs and lateMacs), (iii) almost 100% of regMacs transition into a repMac state and (iv) repMacs retain the original state and do not undergo phenotypic transition.^1^

By performing the analysis at different time points following injury, this protocol allows the identification of alterations in macrophage lineage decisions along the regenerative process. Differences in lineage transitions can be detected by comparing the output of a specific macrophage subset transplant, at two different time points after injury. In a young, healthy animal, an increase in the output of lateMacs is expected from 2 to 3 dpi for all transplanted subpopulations, except for repMacs.^1^

## Quantification and statistical analysis

Following these steps, the number of recorded events for each macrophage subpopulation within the CD45.1^pos^ gate can be obtained as an output. Using these data, the percentage of each final macrophage state relative to the total CD45^pos^ population can be calculated by dividing the number of recorded events for each macrophage state by the total number of CD45.1^pos^ macrophages (sum of all individual macrophage states). These data can be represented in a graph for each subpopulation transplanted, with the x axis displaying each final macrophage state and the y axis showing the percentage of that state relative to the transplanted CD45^pos^ population (see Sousa et al., 2024^1^ for a representative figure of this analysis). A minimum number of CD45.1^pos^ cells should be analyzed to ensure accurate discrimination of final macrophage states. We recommend excluding animals in which the number of CD45.1^pos^ events recovered in the recipient muscle is below 1 × 10^3^ cells. Similarly, if the injury in the recipient muscle is incomplete (assessed as described in step 22), the macrophages will not undergo their phenotypic transition in the expected stereotyped manner, and the data will not be representative. We recommend excluding animals that fail to meet the full injury criteria and halting the protocol beyond step 22.

The analysis described above can be performed in different conditions (e.g. different time points following injury, WT vs knock-out (KO) mice, etc. ) allowing for the identification of factors that affect macrophage lineage decisions. To determine whether the factor in analysis has a significant impact on macrophage lineage decisions, statistical testing can be applied to compare the output in both conditions (e.g., compare the percentage of lateMacs originated at 2 vs 3 dpi, or from WT vs KO mice, for infMacs transplants). For statistical analysis, data sets should be tested first for normal distribution with, e.g., a Shapiro-Wilk test. To assess if differences between groups are significant, a 2-tailed Student’s t test or a two-tailed Mann–Whitney test can be applied for data sets with or without normal distributions, respectively. These data can be represented in a graph, for each subpopulation transplanted and final output state, with the x axis displaying each condition and the y axis showing the percentage of the output state relative to the transplanted CD45^pos^ population (see Sousa et al., 2024^1^ for a representative figure of this analysis comparing macrophage lineage decisions at 2 vs 3dpi).

## Limitations

A few limitations should be considered when implementing this protocol or adapting it to other macrophage subpopulations.

First, although we transplant 1 × 10^5^ macrophages, the majority of the transplanted cells do not survive, and the number of CD45^pos^ macrophages recovered from the recipient muscle can be variable. Moreover, since macrophage subtypes display different survival rates after transplant, when adapting this protocol to a new macrophage population, optimizations may be required to identify the optimal number of transplanted cells that ensures the recovery of sufficient CD45.1^pos^ events for analysis.

Secondly, this protocol has only been tested for phenotypic transitions occurring within a 16 h time frame. If adapted to track lineage decisions over longer periods, the survival rate of the desired macrophage population should be first assessed. Macrophages can die or be eliminated after fulfilling their role during SkM regeneration, however the lifespan of an infiltrating monocyte-derived macrophage in the injured SkM has not yet been determined.

## Troubleshooting

### Problem 1

Number of cells in the single-cell suspension after muscle processing (steps 10 and 22) falls outside the expected range.

### Potential solution

• This may indicate a failed or partial injury. When the injury is not complete, immune infiltration is reduced and this is reflected in a reduction in the number of cells per mg of muscle after processing. To improve the efficiency of the muscle injury (step 1):
  ○ Ensure that 1.2% of BaCl_2_ aliquots are correctly prepared and stocked at −20°C.
  ○ Discard BaCl_2_ aliquots after use to maintain consistency across injuries performed on different days.
  ○ Verify proper intramuscular injection technique, follow the instructions described in this protocol (step 1). Make sure that while injecting the muscle, the needle does not go beyond the muscle.
  ○ If the problem persists, try to inject the defined volume of 1.2% of Barium chloride at two different muscle sites instead of a single site to improve dispersion.
• Alternatively, this may indicate incomplete processing of the muscle tissue. To increase the number and viability of the cells during muscle processing, several strategies can be applied:
  ○ The tissue should be cut into small pieces to maximize the activity of collagenase B during digestion (step 6a).
  ○ During incubation with collagenase B at 37°C, make sure to shake vigorously and periodically (step 7c).
  ○ Prolonging the digesting time decreases cell viability. We recommend limiting the enzymatic digestion to a maximum of 1h and 20 min.
  ○ Samples should be kept on ice or at 4°C throughout the entire procedure to minimize cell death.
  ○ Ensure the pellet is not aspirated during the aspiration steps.

### Problem 2

Low yield of FACS-sorted macrophage subpopulations (step 13) from the donor injured muscles.

### Potential solution

• When setting gates for sorting, consider that macrophage subpopulations vary in size and granularity. Overly strict gates may exclude some populations.
• To reduce debris, filter the samples through the cell strainer cap of the FACS tube or using a cell strainer of 40 μm. If necessary, filter 2 or 3 times. Excessive debris can lead to a lower yield from sorting, as the target cells become rarer events. It can also lead to sample clogging during sorting, impacting the quality and yield of the sorted samples.
• Increase the number of donor mice used in your experiment. The problem may lie in the age or sex of the animals used as donors (e.g., aged mice have reduced numbers of specific macrophage subpopulations^1^).

### Problem 3

The sorting takes an unusually long time (step 13).

### Potential solution

• Pooling all samples from the same condition before sorting can reduce overall sorting time.
• Use methods of purification of immune cells from SkM before FACS. We suggest using a 40% / 80% gradient of Percoll for cell separation. This additional step will reduce the debris and dead cells present in the samples, increasing the proportion of target cells and reducing the sorting time per sample.

### Problem 4

Difficulty in separating cell populations during FACS (step 13) or flow cytometry analysis (step 25).

### Potential solution

• Ensure accurate counting of the total number of cells isolated from muscle and stain them at a defined concentration of 1 × 10^6^ cells per 100 μL of master mix.
• Ensure that single-color controls used for compensation are prepared in the same way as experimental samples, using identical antibody dilutions. Ideally, use cells isolated from an injured muscle (at 2- or 3-dpi), as autofluorescence and cell populations differ between injured and non-injured muscles.
• If using compensation beads for single-stain controls in flow cytometry, consider switching to cells when possible. Cells in single-color controls have the same shape and size as those in the experimental samples and reflect the actual expression levels of markers, which influence fluorescence intensity. This improves automatic compensation matrix calculations performed by BD FACSDiva software in the flow cytometer, minimizing manual adjustments and reducing bias.

### Problem 5

Low number of CD45.1^pos^ cells detected in the recipient muscle after transplantation (step 25).

### Potential solution

• This issue could be related to a decline in the viability of the CD45.1^pos^ cells before transplantation, related to improper handling of the cells during sorting (step 13) and/or preparation of the sorted cells for transplant (step 14). To improve cell viability:
  ○ Place 1.5 mL collection tubes in a cooled (4°C) tube holder during sorting. Keep previously sorted samples on ice while sorting other samples (step 13).
  ○ Keep cells on ice or at 4°C throughout the preparation process (step 14).
  ○ Add a viability dye to the master mix to stain isolated cells from donor mice muscle (step 11). During sorting, use this dye in the gating strategy to select the live cells (step 13).
• Alternatively, this may occur due to the leaking of cell suspension during the intramuscular injection (step 15).
  ○ Try to decrease the volume of saline used to resuspend the cells to 20 μL.
• It is also possible that the conditioning of the recipient mice using clodronate-liposomes (step 2) did not sufficiently reduce the population of macrophages in the muscle before transplantation.
  ○ Ensure correct storage of the clodronate-liposomes at 4°C.
  ○ Homogenize the liposomes thoroughly before loading the syringe.
  ○ Ensure you are applying proper technique during the tail vein injection. Failure of intravenous delivery is indicated by a resistance during injection and the appearance of a whitening around the injection site. This could be due to insufficient dilation of the vein and could be improved by longer exposure to the heat lamp.

## Resource availability

### Lead contact

Further information and requests for resources and reagents should be directed to and will be fulfilled by the lead contact, Joana Neves (joana.neves@gimm.pt).

### Technical contact

Technical questions on executing this protocol should be directed to and will be answered by the technical contact, Neuza S. Sousa (neuza.sousa@gimm.pt).

### Materials availability

This study did not generate new unique reagents.

### Data and code availability

The published article includes all datasets generated or analyzed during this study.

## Acknowledgments

We thank the Flow Cytometry and Rodent facilities of GIMM for their technical support. This study was supported by funding from Fundação para a Ciência e a Tecnologia (PTDC/MED-OUT/8010/2020 and EXPL/MED-OUT/1601/2021), a European Molecular Biology Organization Installation grant (IG4448), la Caixa Foundation (LCF/PR/HR23/52430025), and an ERC grant (immSC-AgingFate, 101126073). N.S.S. (2022.14294.BD) was supported by a fellowship from Fundação para a Ciência e a Tecnologia. J.N. (2021.03843.CEECIND/CP1673/CT0010) and P.S.-V. (2023.09434.CEECIND/CP2857/CT0004) were supported by an employment contract from Fundação para a Ciência e a Tecnologia. Our graphical abstract was created using BioRender.com.

## Author contributions

N.S.S., P.S.-V., and J.N. conceived the protocol and wrote and revised the protocol manuscript. N.S.S. performed the experiments and prepared the figures.

## Declaration of interests

The authors declare no competing interests.
